# The unequal effects of austerity measures between income-groups on the access to healthcare: a quasi-experimental approach

**DOI:** 10.1186/s12939-021-01412-7

**Published:** 2021-03-16

**Authors:** Lore Torfs, Stef Adriaenssens, Susan Lagaert, Sara Willems

**Affiliations:** 1 Department of Public Health and Primary Health Care, Ghent University, C. Heymanslaan 10 (6K3), B-9000 Ghent, Belgium; 2 KU Leuven, Research Centre for Economics (ECON), Warmoesberg 26, B-1000 Brussels, Belgium

**Keywords:** Economic recession, Austerity, Access to healthcare, Unmet medical needs, Inequity in health

## Abstract

**Background:**

The Great Recession, starting in 2008, was characterized by an overall reduction in living standards. This pushed several governments across Europe to restrict expenditures, also in the area of healthcare. These austerity measures are known to have affected access to healthcare, probably unevenly among social groups. This study examines the unequal effects of retrenchment in healthcare expenditures on access to medical care for different income groups across European countries.

**Method:**

Using data of two waves (2008 and 2014) of the European Union Statistics of Income and Living Conditions survey (EU-SILC), a difference-in-differences (DD) approach was used to analyse the overall change in unmet medical needs over time within and between countries. By adding another interaction, the differences in the effects between income quintiles (difference-in-difference-in-differences: DDD) were estimated. To do so, comparisons between two pairs of a treatment and a control case were made: Iceland versus Sweden, and Ireland versus the United Kingdom. These comparisons are made between countries with recessions equal in magnitude, but with different levels of healthcare cuts. This strategy allows isolating the effect of cuts, net of the severity of the recession.

**Results:**

The DD-estimates show a higher increase of unmet medical needs during the Great Recession in the treatment cases (Iceland vs. Sweden: + 3.24 pp.; Ireland vs. the United Kingdom: + 1.15 pp). The DDD-estimates show different results over the two models. In Iceland, the lowest income groups had a higher increase in unmet medical needs. This was not the case in Ireland, where middle-class groups saw their access to healthcare deteriorate more.

**Conclusion:**

Restrictions on health expenditures during the Great Recession caused an increase in self-reported unmet medical needs. The burden of these effects is not equally distributed; in some cases, the lower-income groups suffer most. The case of Ireland, nevertheless, shows that certain policy measures may relatively spare lower-income groups while affecting middle-class income groups more. These results bring in evidence that policies can reduce and even overshoot the general effect of income inequalities on access to healthcare.

**Supplementary Information:**

The online version contains supplementary material available at 10.1186/s12939-021-01412-7.

## Background

One can expect that recessions do not only affect the living standard of populations, but also their access to healthcare. This expected effect may be due to the welfare loss of the population, but also to austerity measures taken by the government. The latter is the focus of this contribution. In particular, we expect that these effects are different between income-groups. This article studies whether a reduction in health expenditures affects access to healthcare differently between income-groups. To do so we compare the effect of budget cuts in different countries on different income groups. Because the budget cuts often follow from the severity of the crisis, we select comparisons between similar countries who suffered at the same level through the Great Recession, but who responded differently in the retrenchment of the public health budgets. This strategy allows documenting whether the effects of budget cuts in healthcare are different between income groups, isolated from the overall effect of the recession. Although several studies document the effect of the Great Recession on unmet medical needs (UMN), none of these disentangle the recession effects as such from the real reduction in health expenditures.

The member states of the World Health Organization committed in 2005 to develop a healthcare system that gives access to healthcare to all people and protects them from financial hardship [1]. This commitment may have contributed to the development of universal coverage in almost all European countries. Despite this universal coverage, a considerable proportion of the citizens experience difficulties in taking up medical care, especially because of financial reasons. Moreover, these difficulties are more common in certain social groups, contributing to inequity in healthcare [2].

In 2008 Europe was hit by an economic crisis called the “Great Recession” because of its great impact on polities and people. It was characterised by the deepest and longest recession after the Great Depression in the 1930s [3]. In many countries, especially in Europe, this Great Recession forced governments to introduce austerity measures in different fields, also in healthcare, due to the increasing deficits in government budgets [4]. Previous research found that public spending on healthcare in Europe tended to fall when there is pressure for cuts in public spending, and this often at a faster pace than other types of government expenditure [5]. As a result, a slower real annual growth rate in per capita health expenditures was noted in the period during and after the crisis in most OECD countries [6]. This was part of an attempt to keep the budget in balance [4, 7]. These reductions in health expenditures often increased out-of-pocket payments (OOP) [4]. The combination with a decline of the purchasing power in many countries, due to soaring unemployment and decreasing wages, made it more difficult for citizens to pay for the rising OOP for medical care [8]. This is expected to affect access to healthcare, measured by an increase of UMN [9].

**Fig. 1:**
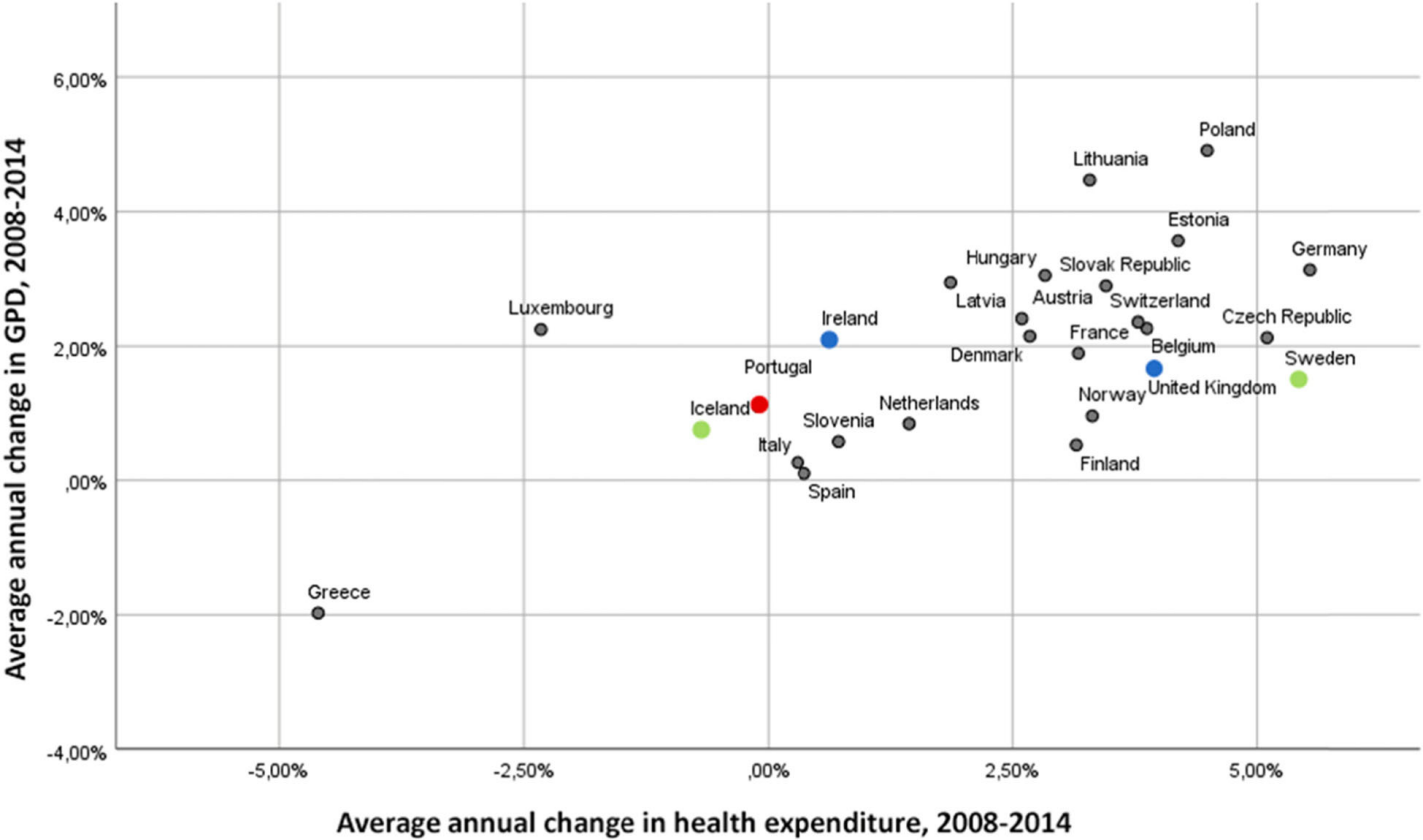
Average annual change in GDP and government health expenditures across countries, 2008–2014. Source: own representation of the OECD Economic References database [10] and Health expenditure and financing database [11]. Average annual change in GDP as a percentage of average annual change between 2008 and 2014 in per capita, PPP, and Average annual change in health expenditure as a percentage of average annual change between 2008 and 2014 in per capita, current prices, current PPP

The relationship between the average annual change in gross domestic product (GDP) and average annual change in health expenditures across countries in the European Union, Iceland, Norway, and Switzerland over the period 2008–2014 is presented in Fig. 1. Overall, the figure illustrates the strong correlation between the severity of the recession and the public health budget trend (*r* = 0.603; *p* < 0.001).

At the same time, it is clear that it still is possible to find countries where the average annual change in GDP was almost the same, while there were strong differences in terms of average annual change in health expenditures. Even more so, some pairs of countries are quite similar in terms of socio-economic structures and institutions, welfare, and even welfare state institutions. The nominal average annual change in GDP over the period 2008–2014 in Sweden (SE) and Iceland (IE) was respectively 1.52% and 0.77%, while the average annual change in health expenditures varied much more (SE: 5.43%; IS: − 0.69%). Similarly, the average annual change in GDP over the period 2008–2014 in the United Kingdom (UK) and the Republic of Ireland (IR) was quite close (UK: 1.66%; IR: 2.09%). The difference between the two countries was that the United Kingdom safeguarded its healthcare spending trends during the crisis, whereas Ireland reduced its health expenditures. In the United Kingdom, the real average annual growth of health expenditure was 3.94% during the period 2008–2014, while it slowed down considerably in Ireland during the same period (0.63%). We aim to isolate the effect of budgetary retrenchment from the severity of the recession. Therefore, it is crucial to learn that at least two pairs of countries exist that are similar in the severity of the recession, and in many other institutional, socio-economic, historical, and geographical aspects.

In parallel with the variation in changes in healthcare spending, the areas in which healthcare policies have been implemented after the Great Recession differ greatly from country to country. Mladovsky et al. [4] categorized the policy measures implemented in the healthcare sector in response to the economic crisis into three groups: policies intended (a) to change the level of contributions for publicly financed healthcare, (b) to affect the volume and quality of publicly financed healthcare and (c) to affect the costs of publicly financed healthcare (see Table 1).

**Table 1:** Implemented policy measure as a response to the Great Recession

Policy measure	Country
**Changing the level of contributions for publicly financed healthcare**	
Cutbacks	**IS**, **IR**, IT, GR, PT, ES
Increasing employee contribution rates	GR, PT, SI
Increasing or introducing user charges	**IR**, CH, CZ, DE, ES, FR, GR, IT, LV, NL, PT, SI
Expanding benefits, targeting low-income groups	**IR**, AT, FR, IT
**Affecting the volume and quality of publicly financed healthcare**	
Changing the scope of coverage	**IR**,EE,, NL, PT
Changing in the population of coverage	**IR**
**Affecting the costs of publicly financed healthcare**	
Reducing the salaries of health professionals	**IS**, **IR**, **UK**, DE, FR, GR, LI, PT, SI.
Changing in provider infrastructure and capital investment	**IS**, **IR**, DE, GR, GR, LI, LV,NL, PT, SI
Centralization of the healthcare organisations (merging hospitals)	**IS**, DE, GR, LV, PT, SI
Reducing the tariffs paid to providers	**IR**, EE, SI

Sources: own representation of Mladovsky, P., et al. [4]

*AT* Austria, *CH* Switzerland, *CZ* Czech Republic, *DE* Denmark, *EE* Estonia, *ES* Spain, *FR* France, *GR* Greece, *IR* Ireland, *IS* Iceland, *LI* Lithuania, *LV* Latvia, *NL* the Netherlands, *PT* Portugal, *SI* Slovenia, *UK* United Kingdom

First, some policies intend to change the level of contributions for publicly financed healthcare. Some countries made cutbacks in the provision of healthcare services, others increased employee contribution rates (either for the general public or for specific population subgroups), and others increased or introduced user charges for health services. Further, some countries reported expanding benefits, targeting low-income groups. These are important policy measures, because user charges play an important role in the threshold for healthcare access, both for low-value care and for high-value care (which is cost-effective). Chaupain-Guillot and Guillot [2] report a positive relationship between the height of UMN and the height of OOP citizens are to pay. Rice et al. [12] also found a link between the level of OOP expenditures and the non-take-up of healthcare when it was considered necessary due to the high costs. They specified that not only the height of the OOP was crucial, but also what citizens were accustomed to paying. Forgoing medical care because of rising OOP is more likely to occur among lower-income individuals [13–16]. This is consistent with the expectation that the relative burden of OOP decreases with the income, resulting in more inequity in access to healthcare.

Second, although some countries modified the coverage scope, they usually left population coverage untouched. Ireland was the only country where changes in population coverage took place for wealthy individuals over the age of 70. Although the population coverage did not change in Greece during the crisis, the rate of uninsured citizens increased, because citizens lost their coverage after 2 years of unemployment until 2014. While a ‘Poverty Booklet’ gave the long-term unemployed, who lived on low benefits, coverage for limited health services until 2006, there was no insurance for them between 2006 and 2014. In June 2014, a new measure gave the uninsured people coverage for prescribed pharmaceuticals and services in emergency departments in public hospitals, as well as for non-emergency hospital care under well-defined conditions [17].

Last, there were also policies intended to affect the costs of publicly financed healthcare. These included the reduction of the salaries of health professionals, as well as changes in provider infrastructure and capital investment. Some countries reorganised the healthcare sector, resulting in centralisation through hospitals merging. Although this centralisation may increase efficiency, it may also have an impact on the accessibility of healthcare.

It can be expected that policy measures, both budget cuts and other measures taken during the Great Recession in Europe have an impact on the accessibility of healthcare. Although several studies report a significant increase in unmet medical needs (UMN), none of these studies made use of a measure of the real reduction in health expenditures [18–22]. Some of them just analysed the evolution in one country by using measurements of UMN before and during the economic crisis [20–22]. Others used the change of GDP and the country’s income inequality to compare the different evolution of UMN during the Great Recession between countries [18].

Also, some of these previous studies allocated the citizens into different groups and reported a differential effect between these groups [18, 20–22]. Elstad [18], who studied the effect of the Great Recession on access to healthcare in all EU-members, categorized people by using the level of income as well as the overall health condition. He documented a stronger increase in UMN in the group he defined as disadvantaged. The other three studies focused just on one country. In Greece, Zavras et al. [22] also used income groups and saw a more marked increase in UMN in the lower-income groups. In contrast, Schneider and Devitt [21] studied the Irish case and reported a higher increase of UMN in the two highest income quartiles. Legido-Quigley et al. [20] allocated the Portuguese people by their employment status and found a greater increase in UMN in the employed than in the unemployed. It can be expected that a differential effect of public expenditures on UMN for distinct income groups is contingent on the policy decisions taken. Scientific attention to the effects of budget cuts on healthcare takeup is important because the underconsumption of healthcare when it is needed can lead to a further deterioration of health [23, 24], and avoidable hospital admissions in the medium term. These negative effects do not only affect life expectancy and the quality of life, but they may also increase the net costs in the longer term [25].

This article aims to study whether a reduction in health expenditures affects access to healthcare differently between income-groups.

Using a difference-in-difference-in-differences (DDD) approach enables us to investigate the extent to which budget cuts affect income groups differentially. Overall, recessions imply a decrease in living standards, which affect their access to healthcare because of the increased relative weight of OOP in households’ budgets. This direct effect is not our central research question, as this effect has been documented quite well in the existing literature. Our research question looks at the indirect impact of the recession, through the ensuing healthcare budget retrenchment. Our central research question is as to whether the latter affects lower-income groups harder than middle-class and high-income groups.

The results of this study can inform policymakers in their decisions on how to deal with future challenges that require cuts in public spending while avoiding the increase of unequal barriers to healthcare, which facilitate health inequity. Thus, it may help to avoid or limit large increases in UMN, or at the very least to reduce inequitable effects for low-income groups.

## Methods

### Data

This study uses individual and household data from the European Union Statistics on Income and Living Conditions program (EU-SILC). The EU-SILC is a harmonized representative population survey on living standards, income, and social inclusion [26]. The survey is conducted each year in the EU countries as well as in Iceland, Norway, and Switzerland. Since the Great Recession started in 2008 with most of the policy measures implemented in 2008–2014, this study builds on the data of the 2008 and the 2014 data waves. This allows us to estimate the impact of the measures introduced.

In the survey, the respondents were asked if they needed medical care but were unable to take it up during the past 12 months. This indicator, “unmet medical needs” (UMN), can be used as a proxy for experienced barriers in access to care. This approach has been adopted in a large number of previous studies [18–20, 27, 28].

Additionally, respondents are asked about the reason for not taking up medical care, choosing between eight options: could not afford care, waiting times, lack of time, travel distance, fear, wait and see, lack of knowledge, or others. A binary outcome variable was constructed indicating the presence of UMN because of cost-related reasons (direct costs, waiting lists, or travel distance). This variable, also used in previous research [18, 28], is likely to reflect difficulties in access to medical care due to situations associated with an economic crisis, such as budget cuts, an insufficient supply of healthcare, higher co-payments, and lack of household economic resources. Income groups were created based on the equivalized income; it is the total income of a household, after tax and other deductions, divided by the equivalized household size. The equivalized household size is based on the modified equivalence scale created by the Organisation for Economic Cooperation and Development (OECD). By doing so, the real living standards of households, net of their size or composition, were used for the analyses.

### Design

To identify whether the impact of budget cuts in the healthcare sector on access to care is different between income-groups, we developed a difference-in-difference-in-differences (DDD) styled regression approach. This regression model adds an extra level to the difference-in-differences (DD) design. The DD is widely used in public health research and policy evaluation, where data are available from different groups, countries, or regions at different times [29]. The technique allows us to estimate effects by comparing a change over time in a treated group (the first difference) with the difference over the same period in a control group (second difference). The difference between the difference in the treated group (the first difference) and the difference in the control group (the second difference) is the difference-in-differences [30, 31]. The addition of an extra interaction term, namely income-group (the third difference), in the DDD allows us to estimate, not only the different changes between countries over time but more specifically whether the effects are different between income-groups.

The proportion of participants who reported UMN due to a lack of means in 2008 is compared with the same proportion in 2014 in the treated country. This difference is compared with the equivalent change in the proportion of participants who experienced UMN due to a lack of means in 2008 and 2014 in the control country. To estimate these differences under the control of control variables, we use linear regressions. One definite advantage of linear regressions is that they allow a comparison of coefficients between different regression estimates. This is the most important reason why we choose these models and not logit or probit regressions, where the comparison between different regression estimates is problematic [32]. Previous research shows that the use of linear probability models is legitimate, as long as the problem of heteroscedasticity is tackled [33]. For this purpose, we estimate cluster-robust standard errors clustered at the level of countries within years.

With this DDD-technique a natural experiment is set up comparing European countries that responded differently in their spending on healthcare, while they suffered from the same recession in order of magnitude. The selection of countries is based on both quantitative (minimizing the difference in change in GDP, maximizing the difference in health expenditure evolution for the period 2008–2014) and qualitative (the type of measures) elements, which were described above (Fig. 1 and Table 1). On top of that, we were able to select pairs that are quite similar in terms of welfare state provisions and the overall institutional and policy-related functioning of the countries. The selection results in 2 comparisons:

1. Treatment: Ireland – control: United Kingdom
2. Treatment: Iceland – control: Sweden

In the two cases, the change in GDP is quite similar between the compared countries, while there is a strong difference in terms of change in health expenditures (see Fig. 1). This allows analysing the impact of budget cuts, isolated from the effects of the Great Recession. In the natural experiment, the cases do not only differ from one another in health expenditure trends but also in a qualitative way: measures taken in Ireland provided greater protection for the lowest income groups [4].

To estimate whether retrenchment in healthcare affects the access to care differentially according to the income-group, a DDD is set up.

The regression equation of the DDD is:

$$ Y={\hat{\beta}}_0+{\hat{\beta}}_1. country+{\hat{\beta}}_2. time+{\hat{\beta}}_3. income+{\hat{\beta}}_4. country. time+{\hat{\beta}}_5. country. income+{\hat{\beta}}_6. time. income+{\hat{\beta}}_7. country. time. income+{\hat{\beta}}_8. control+e $$

We compare pairs of countries between the years 2008 and 2014. Therefore, country and time are dummy variables, indicating whether the observation was in a treated country, post-treatment (in 2014). The equivalized income-groups are divided into quintiles, to analyse the difference between the first quintile (the 20% of the population with the lowest income) with the other groups. This allows us to discover potential non-linear effects, especially to test whether the middles class has evolved differently from the lowest or higher-income quintiles. The interaction term ${\hat{\beta}}_7$ (country*time*income) is of central interest. It reflects the degree to which the change in access to healthcare has increased or decreased in the period 2008–2014 in the different income quintiles compared to the first income quintile. We include a vector of control variables, inspired by earlier research investigating access to healthcare [18–20, 34]. Control variables in the model are age, gender, marital status, urbanization, basic activity, general health, suffering from a chronic illness, and limitations because of health status.

DD(D) techniques start from an equal trends assumption, namely that in the absence of the treatment (in our design: the budget cuts in healthcare), the trends between the two groups would have evolved parallel to one another [30]. Because it is not possible to observe the counterfactual in the real world, one often checks whether the trends were parallel in a period before the treatment. Unfortunately, the EU-SILC has not run long enough to test the parallel trends assumption in a pre-treatment period. We developed several other tests and arguments that support the plausibility of equal trends.

First, some scholars have recently argued estimations become more plausible if the cases are similar in levels *before* the treatment [35]. This indeed seems to be the case in our two pairs. In Sweden (2.4%) and Iceland (1.6%), the reported prevalence of UMN is not that far apart, and the difference is opposite to the post-treatment situation: access to healthcare in Iceland, as measured by UMN, is better before the budget cuts, and worse after. In our main comparison between Ireland (1.7%) and the United Kingdom (1.1%), the pre-treatment differences are particularly small.

The second argument supporting the parallel trends assumption is that, before 2008, EU countries invested in the coordination of health policies through the Open Method of Coordination. This favoured EU-policy in healthcare and other social policy domains, agreed upon in 2000, started to bear some fruit. Scholars seem to argue that the OMC helped to counterbalance potential divergence in policy [36, 37] which would be some reassurance of parallel trends.

Finally, we test an alternative empirical strategy that has been proposed. We tested the equal trends assumption by repeating the analysis on cases that are evenly treated. We, therefore, replicated the regressions for the treated countries (Iceland and Ireland) with a country where both the change in GDP and the change in health expenditures are close to the treated country (Iceland – Portugal, and Ireland – Portugal).

All analyses are conducted in Stata/15.

## Results

### Descriptive analysis

The descriptive analysis of the control variables used in this study can be found in Additional file 1. The results are shown separately for each treatment and control country (Iceland, Sweden, Ireland, the United Kingdom, and Portugal) for both years 2008 and 2014. Table 2 shows the results of the descriptive analysis of the variable of interest, UMN due to causes related to an economic crisis, respectively for 2008 and 2014, separate for each country. In 2008 the highest amount of UMN was reported in Sweden (2,4%), while in 2014 in Sweden (1,4%) the lowest amount of UMN was found. In 2014 the highest amount of UMN was reported in Iceland (4,4%).

**Table 2:** Descriptives - UMN due to situations related to an economic crisis, 2008–2014

		IS		SE		IE		UK		PT	
		n	%	n	%	n	%	n	%	n	%
2008	Unmet medical needs^a^	47	1.6	181	2.4	170	1.7	161	1.1	140	1.4
	No unmet medical needs^b^	2835	98.4	7240	97.6	9940	98.3	14,879	98.9	9952	98.6
	Missing	3732	56.4	7468	50.2	6	0.1	1783	10.6	9	0.1
	Total	6618	100	14,889	100	10,116	100	16,823	100	10,101	100
2014	Unmet medical needs^a^	132	4.4	81	1.4	410	3.9	367	2.1	567	3.9
	No unmet medical needs^b^	2862	95.6	5705	98.6	10,219	96.1	17,524	97.9	14,130	96.1
	Missing	3940	56.8	5491	48.7	0	0.0	14	0.1	4	0.0
	Total	6934	100	11,277	100	10,629	100	17,905	100	14,701	100

Source: Authors’ analysis of EU-SILC 2008 and 2014

*GR* Greece*, AOC* All other countries except Greece, *IS* Iceland, *SE* Sweden, *IE* Ireland, *UK* United Kingdom, *PT* Portugal

^a^Unmet medical needs limited to reasons due to situations related to an economic crisis (due costs, waiting lists, or travel difficulties)

^b^No unmet medical needs or other reasons for unmet medical needs

### Regression analysis

To estimate the effect of budget cuts in healthcare on the accessibility of care in general a difference-in-differences approach was used. Since this is not the main interest of this paper, the results are presented only briefly. In both cases (Iceland compared with Sweden and Ireland compared with the United Kingdom), the UMN increase more in the country that introduced budget cuts in healthcare compared with the countries that did not. In Iceland, the UMN increased more compared to Sweden (3.24 pp), as well as in Ireland compared with the United Kingdom (1.15 pp). The output of the DD is found in Additional file 2.

The core analyses of this paper are the difference-in-difference-in-differences estimations. The DDD tests the impact of reducing health expenditures on access to healthcare in depth. The overall effect of a differential impact of income on UMN, was first estimated by adding equivalized income to the regression as a continuous variable. These results are found in Additional file 2. Table 3 presents only the results of the DDD-test where the equivalized income is separated into income quintiles and analyzed categorically. Hereby, the second, third, fourth, and fifth income quintiles are compared to the lowest income quintile. This approach allows discovering potential non-linear effects. A significant negative effect is found in Iceland for all income quintiles compared to income quintile 1. The threshold for access to healthcare (UMN) thus increases less for those in a higher income quintile. The increase of UMN in Iceland is similar in quintiles 2, 3, and 4 (respectively: − 1.96 pp.; − 1.62 pp.; − 1.83 pp), while the difference in trend is even much more marked in quintile 5 (− 3.51 pp). The results are quite different in Ireland, where UMN increases more in income quintiles 2, 3, and 4 compared to the lowest income group. In income quintile 3 the highest increase in UMN is found compared to income quintile 1 (1.60 pp) over the period 2008–2014. To test the significance of this difference between the income quintiles a chi^2^ tests were carried out. For each year, a separate chi^2^ test was carried out per quintile, using quintile 1 as a reference. This means that 4 chi^2^ tests were performed per year (quintile 1–2, quintile 1–3, quintile 1–4, and quintile 1–5). In 2008, no significant relationship was found between the income quintile and reporting UMN except from the test with quintiles 1 and 5 (people in quintile 5 were significantly less likely to report UMN than those in quintile 1). In contrast, in 2014, a significant relationship was also found in the chi^2^ test with quintile 3 (χ^2^ (1, *n* = 4252) = 7.5; *p* = 0.006). People in income quintile 3 have a higher risk on UMN in 2014 than those in income quintile 1 (Additional file 3). The full output of the DD- and DDD-analyses is attached in Additional file 2.

**Table 3:** DDD-Estimates: the impact of the reduction in health expenditures on UMN over countries and between income groups, 2008–2014

	Iceland		Ireland	
	β	(SE)	Β	(SE)
Income quintile 2	−0.0196**	(0.0005)	0.0029**	(0.0002)
Income quintile 3	−0.0162*	(0.0019)	0.0160**	(0.0003)
Income quintile 4	−0.0183**	(0.0007)	0.0058*	(0.0006)
Income quintile 5 (highest)	−0.0351**	(0.0011)	0.0005	(0.0005)
Control country	Sweden		United Kingdom	
Control variables	yes		yes	
Clustered robust SE (country / year)	yes		yes	

Source: Authors' analysis of EU-SILC 2008 and 2014

Reference category: quintile 1

Unmet medical needs limited to reasons due to situations related to an economic crisis (due costs, waiting lists, or travel difficulties)

*SE* standard error

*p < 0.01; **p < 0.001

## Discussion

This study aims to estimate whether the effects of budget cuts in the healthcare sector on the accessibility of medical care are different between income groups. To estimate these effects, this study evaluates the change in access to healthcare between income-groups over the period 2008–2014, between countries with similar shocks in the business cycle, but differences in the decrease in health expenditures. This approach allows us to analyse whether the effects of budget cuts on access to healthcare are different between income-groups, isolated from the effects of the recession on living standards. The core contribution of this study deals with the expected differential impact of budget cuts by income group. In this respect, the outcome is dramatically different between Iceland, where no specific measures were taken to protect the lowest income groups, and Ireland, which introduced measures intending to protect low-income groups. In Iceland, the lowest income groups were hit especially hard. In contrast, in Ireland, where the introduced measures had the intention to protect the lowest income group, the middle-income group was hit hardest.

This finding shows that the type of measures taken during a recession impacts who is affected by the measure. The equitable distribution of the burden depends on whether or not income-related measures are taken.

The comparison of Iceland versus Sweden and Ireland versus the United Kingdom in a natural experiment allows focusing on the supply-side effects (healthcare budgets) because the effects on the demand side (severity of the recession) were almost similar in treatment and control groups in the period 2008–2014. This isolates the effects of spending on healthcare from the general and direct effects of the Great Recession on people’s living standards. In short, governance matters for an equitable healthcare system. This is an important added value of this research and addresses a gap in previous research. Previous research that also reported an increase of UMN during the Great Recession [18, 20–22, 24], used the business cycle (the Great Recession) as a proxy of reductions in health expenditures; which is imprecise. Furthermore, earlier research only focused on changes through time within countries and does not compare between countries. The DD design allows taking that into account too.

Our central contribution, nevertheless, pertains to the difference in the evolution between income groups. Here, the results for Iceland are different from those from Ireland. In Iceland, higher-income groups are affected less than those in the lowest income group, which is consistent with previous research [18, 22]. By contrast, in Ireland, the middle-class (income quintiles 2, 3, and 4) is more affected. Thus, it is not the most vulnerable low-income group that experienced the greatest increase in UMN. On the contrary, the largest effect is found in income quintile 3. In 2014, there was a significantly higher proportion who reported UMN in quintile 3 than in quintile 1, while this pattern was not present in 2008. The explanation for this outcome can be found in the policy measures Ireland introduced during the recession. In Ireland, some are holders of a medical card, which gives them free access to a general practitioner (GP) and hospital care, and to prescribed medication at a reduced cost (category I). Others have to pay these costs themselves (category II) [38]. In 2014, the number of medical cardholders was nearly 40% [39, 40]. As entitlement to such a medical card depends on the level of income, most beneficiaries are in income quintile 1 and, to a lesser extent, in income quintile 2. As the policy measures implemented by Ireland affected people exclusively from Category II (e.g., increase in user charges and the abolition of automatic entitlement to medical cards for people above 70 years), they were able to guarantee access for low-income groups that were medical cardholders [41]. Our findings confirm the concerns expressed in earlier research about unequal access to care in Ireland, especially for those who are just above the income level to be entitled to a medical card [42]. Also, Schneider and Devitt [21] report a higher increase in UMN during the Great Recession in the higher income groups but no increase in the lower-income groups in Ireland.

These findings demonstrate the importance of research on the impact of healthcare cuts on access to care, isolating these cuts from changes in living standards. In addition, in-depth research is important to understand which groups of the population are most affected by the measures.

### Strengths and limitations

The use of a difference-in-differences (DD) approach makes it possible to set up a stronger causal design since the effect of budget trends can be isolated, net of differences that already existed before the crisis. This is, even more, the case for the difference-in-difference-in-differences design. This allows us to adequately test inequities in healthcare access for different income groups. Thereby the careful selection of treatment and control cases effectively isolates changes in health expenditures, net of the impact of the crisis on living standards. Moreover, the use of multiple cases makes it possible to distinguish between different effects among countries, depending on the type of measures taken by a country. To the best of our knowledge, this more sophisticated set of techniques has not been used before to investigate the differential effect of austerity measures in healthcare on access to care for different income groups.

Despite several strengths, this study has some limitations.

First, difference-in-differences designs are based on a common trend assumption: in the absence of the treatment, the change in both groups remains the same. It is not possible to test this directly. Therefore, this is typically tested for a period where the treatment did not take place. Since data are only available from 2004 or 2005 to 2016 or 2017, the period before and after the crisis is far too short to test the common trend assumption. To meet this limitation, we brought in some logical arguments that in favour of the design. First, the selected comparisons between treatment countries and controls, are similar in levels *before* the treatment (as suggested in the recent literature [35]). In Iceland the small difference is opposite to the post-treatment situation. The pre-treatment difference between Ireland and the United Kingdom is particularly small. Second, social policy scholars see little reason to suspect a divergence in policies before 2008, especially in EU countries. These countries invested considerable effort in the so-called ‘Open Method of Coordination’. OMC probably helped to prevent a potential divergence in policy [36, 37]. Finally, a placebo test was carried out between countries where both the change in wealth (change in GDP) and change in health expenditures over the crisis period are very similar.

A second limitation lies in the variable of interest, self-reported unmet medical needs (UMN). For one thing, the sampling method varies between countries. There are also differences in the nonresponse rate, which is, in particular, higher in Iceland and Sweden. Since there is no other data available, the results of the model with Iceland and Sweden should be interpreted with some caution. Nevertheless, the results of this model are in line with the theoretical expectations and with previous empirical results [9]. In consequence, the model of Ireland, compared to the United Kingdom, is the central case in our contribution. Furthermore, self-reporting may lead to variation in bias between income groups and over time, amongst others, due to adaptation. The subjective relative deprivation theory hypothesizes that, in economic downturns, people adapt their preferences so that those who lose economic resources switch their opinion from ‘cannot afford’ to ‘do not want’ to shield themselves from unrealistic goals [43]. Although this theory has only been tested on consumption goods, it might have an impact on a person’s perception of healthcare needs. This could lead to an underestimation of the results found. A possible solution would be to use objective data in addition to subjective data, such as real take-up of healthcare (GP consults, number of admissions). Since both subjective and objective data have their limitations, combining both data can increase the quality of research, which is in line with the proposals of Thompson et al. [44] Moreover, the variable UMN is taken up in the cross-sectional part of the EU-SILC survey. Because cross-sectional data have certain limitations, the use of panel data is recommended for this purpose [44].

Last, in this study, the effect of a decrease in UMN was measured per income quintile. However, low income is but one dimension of deprivation [45]. Further research can make use of multidimensional approaches to poverty, social exclusion, and deprivation to make a more in-depth analysis.

## Conclusion

The difficulties in access to healthcare (measured as unmet medical needs) increase more strongly in countries where budget cuts in healthcare were higher (e.g., Iceland and Ireland) during the period 2008–2014. We compare these trends with countries that experienced the same change in wealth (change in GDP) but limited their budget cuts in healthcare (e.g., Sweden and the United Kingdom). Important differences in the effect of budget cuts in healthcare can be seen between income groups, contributing to an increase in inequity. In particular, where low-income earners in Iceland are hit harder than the others, this effect did not occur in Ireland. This can be attributed to accompanying measures with the retrenchment, specifically aimed at low-income groups in Ireland, and which were absent in Iceland. These policies can reduce and even overshoot the general effect of income inequalities on access to healthcare. Introducing well-thought-out measures, while guaranteeing equal access to care for as many residents as possible, is important and is recognised as a core objective in the European Union since 2005 [1]. Not taking up necessary medical care has quite strong negative effects on health, well-being, and social and socioeconomic resilience [22]. Unequal access to healthcare leads to an avoidable increase in health inequity.

## Supplementary Information

**Additional file 1:** Descriptives of the control variables.

**Additional file 2:** Output of DD and DDD.

**Additional file 3:** Output of chi^2^-test Ireland, different income quintiles.

## Data Availability

The data that support the findings of this study are available from Eurostat, but restrictions apply to the availability of these data, which were used under license for the current study, and so are not publicly available. Data are, however, available from the authors upon reasonable request and with permission of Eurostat. Supplementary data are available at OECD online.

## Abbreviations

AOC: All other OECD countries except Greece
AT: Austria
CH: Switzerland
CZ: Czech Republic
DE: Denmark
DD: Difference-in-differences
DDD: Difference-in-difference-in-differences
EE: Estonia
ES: Spain
EU-SILC: European Union Statistics on Income and Living Conditions
FR: France
GDP: Gross domestic product
GR: Greece
IR: Ireland
IS: Iceland
LI: Lithuania
LV: Latvia
NL: the Netherlands
OECD: Organisation for Economic Co-operation and Development
OOP: Out-of-pocket payment
pp.: Percent points
PT: Portugal
SE: Sweden
SI: Slovenia
UMN: Unmet medical need
UK: United Kingdom

## References

[1] World Health Organization (2010). Health systems financing: the path to universal coverage. World health report 2010.

[2] Chaupain-Guillot S, Guillot O (2015). Health system characteristics and unmet care needs in Europe: an analysis based on EU-SILC data. Eur J Health Econ.

[3] Redbird B, Grusky DB (2016). Distributional effects of the great recession: where has all the sociology gone?. Annu Rev Sociol.

[4] Mladovsky P, Srivastava D, Cylus J, Karanikolos M, Evetovits T, Thomson S (2012). Health policy responses to the financial crisis in Europe.

[5] Cylus J, Mladovsky P, McKee M (2012). Is there a statistical relationship between economic crises and changes in government health expenditure growth? An analysis of twenty-four European countries. Health Serv Res.

[6] OECD. Society at a glance 2019. Paris: OECD Publishing; 2019.

[7] Hou X, Velényi EV, Yazbeck AS, Iunes RF, Smith O. Learning from economic downturns: how to better assess, track, and mitigate the impact on the health sector. Washington, DC: The World Bank; 2013.

[8] OECD, European Union (2014). Health at a glance.

[9] Allin S, Masseria C (2009). Unmet need as an indicator of health care access. Eurohealth..

[10] Economic references [database on the Internet]. 2016. Available from: https://www.oecd-ilibrary.org/content/data/data-00548-en.

[11] Health expenditure indicators [database on the Internet]. 2014. Available from: https://www.oecd-ilibrary.org/content/data/data-00349-en.

[12] Rice T, Quentin W, Anell A, Barnes AJ, Rosenau P, Unruh LY (2018). Revisiting out-of-pocket requirements: trends in spending, financial access barriers, and policy in ten high-income countries. BMC Health Serv Res.

[13] Gemmill MC, Thomson S, Mossialos E (2008). What impact do prescription drug charges have on efficiency and equity? Evidence from high-income countries. Int J Equity Health.

[14] Lohr KN, Brook RH, Kamberg CJ, Goldberg GA, Leibowitz A, Keesey J (1986). Use of medical care in the RAND health insurance experiment: diagnosis-and service-specific analyses in a randomized controlled trial. Med Care.

[15] Manning WG, Newhouse JP, Duan N, Keeler EB, Leibowitz A. Health insurance and the demand for medical care: evidence from a randomized experiment. Am Econ Rev. 1987:251–77. 10284091

[16] Newhouse JP. Free for all?: lessons from the RAND health insurance experiment. Cambridge: Harvard University Press; 1993.

[17] Eurofound (2014). Access to healthcare in times of crisis.

[18] Elstad JI (2016). Income inequality and foregone medical care in Europe during the great recession: multilevel analyses of EU-SILC surveys 2008–2013. Int J Equity Health.

[19] Karanikolos M, Gordeev VS, Mackenbach JP, McKee M (2016). Access to care in the Baltic States: did crisis have an impact?. Eur J Pub Health.

[20] Legido-Quigley H, Karanikolos M, Hernandez-Plaza S, de Freitas C, Bernardo L, Padilla B (2016). Effects of the financial crisis and troika austerity measures on health and health care access in Portugal. Health Policy.

[21] Schneider SM, Devitt C (2018). Accessing healthcare in times of economic growth and economic downturn: evidence from Ireland. J Eur Soc Policy.

[22] Zavras D, Zavras AI, Kyriopoulos I-I, Kyriopoulos J (2016). Economic crisis, austerity and unmet healthcare needs: the case of Greece. BMC Health Serv Res.

[23] Peters DH, Garg A, Bloom G, Walker DG, Brieger WR, Hafizur RM (2008). Poverty and access to health care in developing countries. Ann N Y Acad Sci.

[24] Wagstaff A (2002). Poverty and health sector inequalities. Bull World Health Organ.

[25] Weissman JS, Gatsonis C, Epstein AM (1992). Rates of avoidable hospitalization by insurance status in Massachusetts and Maryland. Jama..

[26] Iacovou M, Kaminska O, Levy H. Using EU-SILC data for cross-national analysis: strengths, problems and recommendations. ISER working paper series, 2012.

[27] Kentikelenis A, Karanikolos M, Reeves A, McKee M, Stuckler D (2014). Greece's health crisis: from austerity to denialism. Lancet.

[28] Rodrigues R, Zolyomi E, Kalavrezou N, Matsaganis M. The impact of the financial crisis on unmet needs for healthcare. Employment, Social Affairs & Inclusion, European Commission Brussels; 2013.

[29] Wing C, Simon K, Bello-Gomez RA (2018). Designing difference in difference studies: best practices for public health policy research. Annu Rev Public Health.

[30] Angrist J, Pischke J-S (2009). Mostly harmless econometrics. An empiricist's companion.

[31] Gertler PJ, Martinez S, Premand P, Rawlings LB, Vermeersch CMJ (2016). Impact evaluation in practice. Second edition ed.

[32] Karlson KB, Holm A, Breen R (2012). Comparing regression coefficients between same-sample nested models using logit and probit: a new method. Sociol Methodol.

[33] Mood C (2010). Logistic regression: why we cannot do what we think we can do, and what we can do about it. Eur Sociol Rev.

[34] Elstad JI (2017). Dental care coverage and income-related inequalities in foregone dental care in Europe during the great recession. Community Dent Oral Epidemiol.

[35] Kahn-Lang A, Lang K. The promise and pitfalls of differences-in-differences: reflections on 16 and pregnant and other applications. J Bus Econ Stat. 2019:1–14.

[36] De La Rosa S (2018). The OMC processes in the health care field: what does coordination really mean?. Eur Papers J Law Integration.

[37] Vanhercke B, Vanhercke B, Ghailani D, Spasova S, Pochet P (2020). From the Lisbon strategy to the European pillar of social rights: the many lives of the social open method of coordination. Social policy in the European Union 1999–2019: the long and winding road.

[38] Layte R, Nolan A, Nolan B. Poor prescriptions: poverty and access to community health services. Dublin: Combat Poverty Agency; 2007.

[39] Burke SA, Normand C, Barry S, Thomas S (2016). From universal health insurance to universal healthcare? The shifting health policy landscape in Ireland since the economic crisis. Health Policy.

[40] Government of Ireland. Health in Ireland: Key Trends 2018. Dublin: Department of Health, Epidemiology Unit; 2018.

[41] Thomson S (2014). Health system responses to financial pressures in Ireland.

[42] Brick A, Nolan A, O’Reilly J, Smith S (2010). Resource allocation, financing and sustainability in health care. Evidence for the Expert Group on Resource Allocation and Financing in the Health Sector.

[43] Halleröd B (2006). Sour grapes: relative deprivation, adaptive preferences and the measurement of poverty. J Soc Policy.

[44] Thompson K, van Ophem J, Wagemakers A (2019). Studying the impact of the Eurozone’s great recession on health: methodological choices and challenges. Econ Hum Biol.

[45] Haughton J, Khandker SR (2009). Handbook on poverty and inequality.

