# Supplementary material for: The unequal effects of austerity measures between income-groups on the access to healthcare: a quasi-experimental approach
Source: Int J Equity Health. 2021 Mar 16;20:79. doi: 10.1186/s12939-021-01412-7 (PMC7962334; doi:10.1186/s12939-021-01412-7)
Supplement: Supplementary file 2 — Additional file 2: Output of DD and DDD. [file 12939_2021_1412_MOESM2_ESM.pdf]

```
                                                  ___  ____  ____  ____  ____(R)
                                                 /__    /   ____/   /   ____/
                                                ___/   /   /___/   /   /___/
                                                  Statistics/Data Analysis

                                                    User: R 102 113
-----------------------------------------------------------------------------------------
      name:  <unnamed>
       log:  C:\Users\u0002299\Documents\1. Onderzoek & papers\2020 EU SILC\2020 12 27 R 102 11
  log type:  smcl
 opened on:  27 Dec 2020, 17:30:17

1 . do "C:\Users\u0002299\AppData\Local\Temp\STD3060_000000.tmp"

2 . * Model 102
3 . regress UMN_reason_R T_YEAR T_IS T_ISYR PER_INCOME AGE_R ///
  > Sex Marital_R Chronic_illness Limitation_health_R URB2 URB3 BAC2 BAC3 BAC4 GHE1 GHE2 ///
  > , vce(cluster ROBUST)

  Linear regression                               Number of obs     =         18,841
                                                  F(2, 3)           =              .
                                                  Prob > F          =              .
                                                  R-squared         =         0.0303
                                                  Root MSE          =         .14796

                                         (Std. Err. adjusted for      4 clusters in ROBUST)
  -------------------------------------------------------------------------------------
                      |               Robust
         UMN_reason_R |      Coef.   Std. Err.      t    P>|t|     [95% Conf. Interval]
  --------------------+----------------------------------------------------------------
               T_YEAR |  -.0083294   .0014902    -5.59   0.011      -.013072   -.0035868
                 T_IS |  -.0077428   .0010448    -7.41   0.005      -.011068   -.0044177
               T_ISYR |   .0323636   .0019279    16.79   0.000      .0262282    .0384989
           PER_INCOME |  -.0001932   .0000622    -3.11   0.053      -.000391    4.64e-06
                AGE_R |  -.0003558   .0001928    -1.85   0.162     -.0009693    .0002578
                  Sex |   .0018342    .002445     0.75   0.508     -.0059468    .0096152
            Marital_R |   .0028225   .0021969     1.28   0.289     -.0041688    .0098139
      Chronic_illness |   .0104122   .0023084     4.51   0.020      .0030657    .0177587
  Limitation_health_R |   .0194876   .0076932     2.53   0.085     -.0049955    .0439707
                 URB2 |  -.0001594   .0036765    -0.04   0.968     -.0118595    .0115408
                 URB3 |  -.0037615   .0041511    -0.91   0.432     -.0169722    .0094493
                 BAC2 |   .0368974   .0117705     3.13   0.052     -.0005615    .0743563
                 BAC3 |  -.0117938   .0123329    -0.96   0.409     -.0510427     .027455
                 BAC4 |  -.0007277   .0091857    -0.08   0.942     -.0299607    .0285053
                 GHE1 |   .0181866   .0036828     4.94   0.016      .0064662    .0299071
                 GHE2 |   .0558763   .0176743     3.16   0.051     -.0003711    .1121238
                _cons |   .0392474   .0095412     4.11   0.026      .0088831    .0696117
  -------------------------------------------------------------------------------------


4 .
5 . * Model 103
6 . regress UMN_reason_R T_YEAR T_IE T_IEYR PER_INCOME AGE_R ///
  > Sex Marital_R Chronic_illness Limitation_health_R URB2 URB3 BAC2 BAC3 BAC4 GHE1 GHE2 ///
  > , vce(cluster ROBUST)

  Linear regression                               Number of obs     =         49,507
                                                  F(2, 3)           =              .
                                                  Prob > F          =              .
                                                  R-squared         =         0.0152
                                                  Root MSE          =          .1426

                                         (Std. Err. adjusted for      4 clusters in ROBUST)
  -------------------------------------------------------------------------------------
                      |               Robust
         UMN_reason_R |      Coef.   Std. Err.      t    P>|t|     [95% Conf. Interval]
  --------------------+----------------------------------------------------------------
               T_YEAR |   .0093571   .0003943    23.73   0.000      .0081021     .010612
                 T_IE |   .0082665   .0014765     5.60   0.011      .0035676    .0129653
               T_IEYR |   .0114898   .0005057    22.72   0.000      .0098806    .0130991
           PER_INCOME |  -.0000317   .0000538    -0.59   0.597     -.0002027    .0001394
                AGE_R |  -.0001453   .0000614    -2.36   0.099     -.0003408    .0000502
                  Sex |  -.0049619    .001712    -2.90   0.063     -.0104101    .0004864
            Marital_R |  -.0012629   .0036518    -0.35   0.752     -.0128847    .0103589
      Chronic_illness |    .009245   .0026971     3.43   0.042      .0006615    .0178285
  Limitation_health_R |    .010037    .002214     4.53   0.020      .0029911    .0170828
                 URB2 |  -.0009476    .001254    -0.76   0.505     -.0049385    .0030434
                 URB3 |  -.0043537   .0029788    -1.46   0.240     -.0138336    .0051262
                 BAC2 |   .0044309   .0070079     0.63   0.572     -.0178712    .0267331
                 BAC3 |  -.0150976   .0055915    -2.70   0.074     -.0328921     .002697
```

	Coef.	Std. Err.	t	P>|t|	[95% Conf.	Interval]
BAC4	-.0100469	.0046358	-2.17	0.119	-.0248001	.0047064
GHE1	.0139571	.0059689	2.34	0.101	-.0050388	.0329529
GHE2	.0344529	.0080143	4.30	0.023	.0089477	.059958
_cons	.0191343	.0078559	2.44	0.093	-.0058667	.0441353

```
.
. * Model 104
. regress UMN_reason_R T_YEAR P_IS P_ISYR PER_INCOME AGE_R ///
> Sex Marital_R Chronic_illness Limitation_health_R URB2 URB3 BAC2 BAC3 BAC4 GHE1 GHE2 ///
> , vce(cluster ROBUST)
```

Linear regression

Number of obs	=	30,562
F(2, 3)	=	.
Prob > F	=	.
R-squared	=	0.0287
Root MSE	=	.16513

(Std. Err. adjusted for **4** clusters in ROBUST)

UMN_reason_R	Coef.	Robust Std. Err.	t	P>|t|	[95% Conf.	Interval]
T_YEAR	.0238659	.0004238	56.31	0.000	.0225171	.0252146
P_IS	.0119565	.0025682	4.66	0.019	.0037834	.0201297
P_ISYR	.0015002	.0012712	1.18	0.323	-.0025452	.0055456
PER_INCOME	-.0004453	.0001332	-3.34	0.044	-.0008691	-.0000215
AGE_R	-.0000223	.0000962	-0.23	0.831	-.0003284	.0002837
Sex	-.0067423	.0023749	-2.84	0.066	-.0143002	.0008155
Marital_R	.0039818	.0017344	2.30	0.105	-.0015379	.0095015
Chronic_illness	-.0012114	.0037938	-0.32	0.770	-.0132849	.0108622
Limitation_health_R	.0195933	.0046511	4.21	0.024	.0047913	.0343953
URB2	-.0020591	.0050967	-0.40	0.713	-.0182792	.014161
URB3	-.005164	.001963	-2.63	0.078	-.0114112	.0010831
BAC2	.01227	.0039074	3.14	0.052	-.0001651	.0247051
BAC3	-.0225987	.0062219	-3.63	0.036	-.0423994	-.002798
BAC4	-.003589	.0052776	-0.68	0.545	-.0203845	.0132065
GHE1	.0238074	.0068926	3.45	0.041	.0018719	.0457429
GHE2	.0348543	.0116032	3.00	0.057	-.0020723	.0717808
_cons	.0251838	.0048716	5.17	0.014	.0096803	.0406873

```
.
. * Model 105
. regress UMN_reason_R T_YEAR P_IE P_IEYR PER_INCOME AGE_R ///
> Sex Marital_R Chronic_illness Limitation_health_R URB2 URB3 BAC2 BAC3 BAC4 GHE1 GHE2 ///
> , vce(cluster ROBUST)
```

Linear regression

Number of obs	=	45,318
F(2, 3)	=	.
Prob > F	=	.
R-squared	=	0.0220
Root MSE	=	.16331

(Std. Err. adjusted for **4** clusters in ROBUST)

UMN_reason_R	Coef.	Robust Std. Err.	t	P>|t|	[95% Conf.	Interval]
T_YEAR	.0245197	.0005157	47.54	0.000	.0228784	.026161
P_IE	.0169231	.0033464	5.06	0.015	.0062734	.0275728
P_IEYR	-.0034692	.0004825	-7.19	0.006	-.0050049	-.0019335
PER_INCOME	-.0003282	.0001408	-2.33	0.102	-.0007762	.0001198
AGE_R	-.0001385	.0000919	-1.51	0.229	-.0004309	.0001538
Sex	-.0080309	.0017578	-4.57	0.020	-.013625	-.0024369
Marital_R	-.0012191	.0038611	-0.32	0.773	-.0135069	.0110687
Chronic_illness	.0006679	.0055528	0.12	0.912	-.0170037	.0183395
Limitation_health_R	.0149048	.0025849	5.77	0.010	.0066786	.0231309
URB2	-.0035803	.0034816	-1.03	0.379	-.0146603	.0074997
URB3	-.008201	.0009135	-8.98	0.003	-.0111084	-.0052937
BAC2	.004644	.0064315	0.72	0.522	-.015824	.0251119
BAC3	-.0214509	.0055586	-3.86	0.031	-.0391407	-.003761
BAC4	-.0144041	.0049069	-2.94	0.061	-.0300202	.0012119

GHE1	.0255182	.0053955	4.73	0.018	.0083473	.0426892
GHE2	.0402871	.0107896	3.73	0.033	.0059498	.0746244
_cons	.0304914	.0032012	9.52	0.002	.0203037	.0406791

```
13 .
14 . * Model 107
15 . regress UMN_reason_R T_YEAR T_IS T_ISYR KWIN2 KWIN3 KWIN4 KWIN5 T_ISKWIN2 T_ISKWIN3 T_ISKWIN4 T_ISKWI
   > AGE_R Sex Marital_R Chronic_illness Limitation_health_R URB2 URB3 BAC2 BAC3 BAC4 GHE1 GHE2 ///
   > T_ISYRKWIN2 T_ISYRKWIN3 T_ISYRKWIN4 T_ISYRKWIN5, vce(cluster ROBUST)
```

Linear regression

Number of obs	=	18,841
F(2, 3)	=	.
Prob > F	=	.
R-squared	=	0.0315
Root MSE	=	.14791

(Std. Err. adjusted for 4 clusters in ROBUST)

UMN_reason_R	Coef.	Robust Std. Err.	t	P>|t|	[95% Conf.	Interval]
T_YEAR	-.0081045	.0014555	-5.57	0.011	-.0127364	-.0034725
T_IS	-.0023653	.0042094	-0.56	0.613	-.0157615	.0110309
T_ISYR	.0489625	.0016282	30.07	0.000	.0437807	.0541443
KWIN2	-.0011269	.0071172	-0.16	0.884	-.0237769	.021523
KWIN3	-.004789	.0058949	-0.81	0.476	-.0235492	.0139712
KWIN4	-.0051139	.0049168	-1.04	0.375	-.0207612	.0105335
KWIN5	-.0088873	.0051326	-1.73	0.182	-.0252215	.0074468
T_ISKWIN2	-.0028317	.00643	-0.44	0.689	-.0232948	.0176313
T_ISKWIN3	-.0116501	.0046799	-2.49	0.089	-.0265437	.0032436
T_ISKWIN4	-.0067644	.0035255	-1.92	0.151	-.017984	.0044553
T_ISKWIN5	-.006588	.0036619	-1.80	0.170	-.018242	.0050659
AGE_R	-.0003681	.000193	-1.91	0.153	-.0009824	.0002461
Sex	.0018453	.0024298	0.76	0.503	-.0058874	.009578
Marital_R	.0029715	.0022267	1.33	0.274	-.0041147	.0100577
Chronic_illness	.0104084	.0022837	4.56	0.020	.0031405	.0176763
Limitation_health_R	.0195778	.0078209	2.50	0.087	-.0053119	.0444675
URB2	-.0006187	.0033809	-0.18	0.866	-.0113782	.0101407
URB3	-.0035459	.0041031	-0.86	0.451	-.0166039	.0095121
BAC2	.0378639	.0117631	3.22	0.049	.0004285	.0752994
BAC3	-.0111296	.0120471	-0.92	0.424	-.0494688	.0272096
BAC4	-.0006399	.0093985	-0.07	0.950	-.0305502	.0292704
GHE1	.0183551	.0037955	4.84	0.017	.006276	.0304341
GHE2	.0559581	.0177137	3.16	0.051	-.0004148	.1123309
T_ISYRKWIN2	-.0195746	.0004622	-42.35	0.000	-.0210456	-.0181037
T_ISYRKWIN3	-.0161957	.0018731	-8.65	0.003	-.0221568	-.0102346
T_ISYRKWIN4	-.0182579	.0007181	-25.43	0.000	-.0205432	-.0159726
T_ISYRKWIN5	-.0351404	.0010723	-32.77	0.000	-.0385529	-.031728
_cons	.0338372	.0152125	2.22	0.113	-.0145758	.0822503

```
16 .
17 . * Model 108
18 . regress UMN_reason_R T_YEAR T_IE T_IEYR KWIN2 KWIN3 KWIN4 KWIN5 T_IEKWIN2 T_IEKWIN3 T_IEKWIN4 T_IEKWI
   > AGE_R Sex Marital_R Chronic_illness Limitation_health_R URB2 URB3 BAC2 BAC3 BAC4 GHE1 GHE2 ///
   > T_IEYRKWIN2 T_IEYRKWIN3 T_IEYRKWIN4 T_IEYRKWIN5, vce(cluster ROBUST)
```

Linear regression

Number of obs	=	49,507
F(2, 3)	=	.
Prob > F	=	.
R-squared	=	0.0166
Root MSE	=	.14252

(Std. Err. adjusted for 4 clusters in ROBUST)

UMN_reason_R	Coef.	Robust Std. Err.	t	P>|t|	[95% Conf.	Interval]
T_YEAR	.0094468	.0003368	28.05	0.000	.0083749	.0105188
T_IE	.008037	.0023207	3.46	0.041	.0006515	.0154225
T_IEYR	.0063405	.00071	8.93	0.003	.0040809	.0086001
KWIN2	-.0024363	.0008559	-2.85	0.065	-.0051603	.0002876
KWIN3	-.0005937	.0027968	-0.21	0.845	-.0094943	.0083069
KWIN4	-.0000517	.0012575	-0.04	0.970	-.0040535	.0039502
KWIN5	.0011885	.0024478	0.49	0.661	-.0066014	.0089785
T_IEKWIN2	.0068826	.0005083	13.54	0.001	.0052649	.0085002
T_IEKWIN3	.0059103	.002767	2.14	0.122	-.0028956	.0147161
T_IEKWIN4	.0016287	.0009473	1.72	0.184	-.001386	.0046434
T_IEKWIN5	-.0120262	.0028381	-4.24	0.024	-.0210584	-.0029941
AGE_R	-.0001485	.0000579	-2.56	0.083	-.0003329	.0000359
Sex	-.005071	.0017456	-2.91	0.062	-.0106261	.0004842
Marital_R	-.0010777	.0035231	-0.31	0.780	-.0122897	.0101342
Chronic_illness	.0090424	.0025268	3.58	0.037	.0010009	.0170839
Limitation_health_R	.0100294	.0022913	4.38	0.022	.0027376	.0173212
URB2	-.0015705	.0013149	-1.19	0.318	-.0057551	.0026141
URB3	-.0050557	.0032308	-1.56	0.216	-.0153376	.0052261
BAC2	.0042478	.0068648	0.62	0.580	-.0175992	.0260947
BAC3	-.0147351	.005517	-2.67	0.076	-.0322926	.0028224
BAC4	-.0102569	.0044551	-2.30	0.105	-.0244352	.0039213
GHE1	.0138925	.0058736	2.37	0.099	-.0047999	.0325849
GHE2	.0348969	.0081124	4.30	0.023	.0090796	.0607141
T_IEYRKWIN2	.0029456	.0001913	15.39	0.001	.0023367	.0035546
T_IEYRKWIN3	.0160247	.0003276	48.92	0.000	.0149821	.0170672
T_IEYRKWIN4	.0057702	.0006003	9.61	0.002	.0038598	.0076807
T_IEYRKWIN5	.000524	.0004674	1.12	0.344	-.0009635	.0020115
_cons	.0183242	.0060091	3.05	0.055	-.0007994	.0374477

```
19 .
20 . * Model 112
21 . regress UMN_reason_R T_YEAR T_IS T_ISYR PER_INCOME T_ISIN3 T_YRIN3 ///
   > AGE_R Sex Marital_R Chronic_illness Limitation_health_R URB2 URB3 BAC2 BAC3 BAC4 GHE1 GHE2 ///
   > T_ISYRIN3, vce(cluster ROBUST)
```

Linear regression

Number of obs	=	18,841
F(2, 3)	=	.
Prob > F	=	.
R-squared	=	0.0313
Root MSE	=	.14789

(Std. Err. adjusted for 4 clusters in ROBUST)

UMN_reason_R	Coef.	Robust Std. Err.	t	P>|t|	[95% Conf.	Interval]
T_YEAR	-.0122208	.0016633	-7.35	0.005	-.0175141	-.0069275
T_IS	-.0048895	.0005469	-8.94	0.003	-.00663	-.003149
T_ISYR	.0524326	.0019282	27.19	0.000	.0462963	.058569
PER_INCOME	-.0001436	.0000312	-4.60	0.019	-.000243	-.0000441
T_ISIN3	-.0000586	.0000137	-4.29	0.023	-.0001021	-.0000151
T_YRIN3	.0000836	6.22e-06	13.43	0.001	.0000638	.0001034
AGE_R	-.0003603	.0001895	-1.90	0.153	-.0009635	.0002428
Sex	.0019445	.0023791	0.82	0.474	-.0056268	.0095157
Marital_R	.0030197	.0023041	1.31	0.281	-.004313	.0103524
Chronic_illness	.0104337	.0022569	4.62	0.019	.0032512	.0176162
Limitation_health_R	.0195995	.0078275	2.50	0.087	-.0053111	.0445101
URB2	-.0007126	.0033072	-0.22	0.843	-.0112376	.0098124
URB3	-.0037495	.0041708	-0.90	0.435	-.0170227	.0095237
BAC2	.0375291	.0118687	3.16	0.051	-.0002422	.0753005
BAC3	-.011288	.0119522	-0.94	0.415	-.0493253	.0267494
BAC4	-.0005884	.0092031	-0.06	0.953	-.0298767	.0286999
GHE1	.0183847	.0037407	4.91	0.016	.0064802	.0302892
GHE2	.0558843	.017643	3.17	0.051	-.0002635	.1120321
T_ISYRIN3	-.0004244	8.14e-06	-52.11	0.000	-.0004504	-.0003985
_cons	.0367384	.0129774	2.83	0.066	-.0045617	.0780384

```
22 .
23 . * Model 113
24 . regress UMN_reason_R T_YEAR T_IE T_IEYR PER_INCOME T_IEIN3 T_YRIN3 ///
   > AGE_R Sex Marital_R Chronic_illness Limitation_health_R URB2 URB3 BAC2 BAC3 BAC4 GHE1 GHE2 ///
   > T_IEYRIN3, vce(cluster ROBUST)
```

```
Linear regression                               Number of obs     =     49,507
                                                F(2, 3)           =          .
                                                Prob > F          =          .
                                                R-squared         =     0.0154
                                                Root MSE          =     .14259

                                    (Std. Err. adjusted for 4 clusters in ROBUST)
```

UMN_reason_R	Coef.	Robust Std. Err.	t	P>|t|	[95% Conf.	Interval]
T_YEAR	.0058914	.0006073	9.70	0.002	.0039586	.0078242
T_IE	.0142492	.0021238	6.71	0.007	.0074902	.0210081
T_IEYR	.0135464	.0010938	12.38	0.001	.0100655	.0170273
PER_INCOME	-.0000126	.0000205	-0.61	0.582	-.0000777	.0000525
T_IEIN3	-.0001133	.0000146	-7.76	0.004	-.0001598	-.0000668
T_YRIN3	.0000706	7.25e-06	9.75	0.002	.0000476	.0000937
AGE_R	-.0001549	.0000591	-2.62	0.079	-.0003431	.0000333
Sex	-.0050693	.0017686	-2.87	0.064	-.0106976	.000559
Marital_R	-.001331	.0036534	-0.36	0.740	-.0129579	.0102959
Chronic_illness	.0091048	.0026629	3.42	0.042	.0006302	.0175794
Limitation_health_R	.0100428	.0022638	4.44	0.021	.0028385	.0172471
URB2	-.0012567	.0013452	-0.93	0.419	-.0055377	.0030243
URB3	-.0048237	.0030754	-1.57	0.215	-.0146111	.0049637
BAC2	.0039959	.0072896	0.55	0.622	-.019203	.0271947
BAC3	-.0147398	.0055734	-2.64	0.077	-.0324769	.0029973
BAC4	-.0104565	.0046915	-2.23	0.112	-.0253869	.0044739
GHE1	.0140046	.0060049	2.33	0.102	-.0051057	.033115
GHE2	.035078	.0082408	4.26	0.024	.0088522	.0613039
T_IEYRIN3	-.0000431	.0000127	-3.40	0.042	-.0000834	-2.76e-06
_cons	.0188691	.0061525	3.07	0.055	-.000711	.0384491

```
25 .
26 . log close
         name:  <unnamed>
          log:  C:\Users\u0002299\Documents\1. Onderzoek & papers\2020 EU SILC\2020 12 27 R 102 11
     log type:  smcl
    closed on:  27 Dec 2020, 17:30:55
```
