# Supplementary material for: The unequal effects of austerity measures between income-groups on the access to healthcare: a quasi-experimental approach
Source: Int J Equity Health. 2021 Mar 16;20:79. doi: 10.1186/s12939-021-01412-7 (PMC7962334; doi:10.1186/s12939-021-01412-7)
Supplement: Supplementary file 3 — Additional file 3: Output of chi2-test Ireland, different income quintiles. [file 12939_2021_1412_MOESM3_ESM.pdf]

Crosstabs

PRE08 POST14 = 2008

Case Processing Summary[a]

	Cases					
	Valid		Missing		Total	
	N	Percent	N	Percent	N	Percent
KWIN2 * UMN_reason_R	4043	40,0%	6073	60,0%	10116	100,0%

a. PRE08 POST14 = 2008

KWIN2 * UMN_reason_R Crosstabulation[a]

			UMN_reason_R		Total
			0	1	
KWIN2	,00	Count	1983	35	2018
		% within KWIN2	98,3%	1,7%	100,0%
		% within UMN_reason_R	50,0%	44,3%	49,9%
		% of Total	49,0%	0,9%	49,9%
	1,00	Count	1981	44	2025
		% within KWIN2	97,8%	2,2%	100,0%
		% within UMN_reason_R	50,0%	55,7%	50,1%
		% of Total	49,0%	1,1%	50,1%
Total		Count	3964	79	4043
		% within KWIN2	98,0%	2,0%	100,0%
		% within UMN_reason_R	100,0%	100,0%	100,0%
		% of Total	98,0%	2,0%	100,0%

a. PRE08 POST14 = 2008

Chi-Square Tests[a]

	Value	df	Asymptotic Significance (2-sided)	Exact Sig. (2-sided)	Exact Sig. (1-sided)
Pearson Chi-Square	1,014[b]	1	,314		
Continuity Correction[c]	,798	1	,372		
Likelihood Ratio	1,016	1	,313		
Fisher's Exact Test				,364	,186
Linear-by-Linear Association	1,014	1	,314		
N of Valid Cases	4043				

a. PRE08 POST14 = 2008

b. 0 cells (,0%) have expected count less than 5. The minimum expected count is 39,43.

c. Computed only for a 2x2 table

PRE08 POST14 = 2014

Case Processing Summary[a]

	Cases					
	Valid		Missing		Total	
	N	Percent	N	Percent	N	Percent
KWIN2 * UMN_reason_R	4251	40,0%	6378	60,0%	10629	100,0%

a. PRE08 POST14 = 2014

KWIN2 * UMN_reason_R Crosstabulation[a]

			UMN_reason_R		Total
			0	1	
KWIN2	,00	Count	2049	77	2126
		% within KWIN2	96,4%	3,6%	100,0%
		% within UMN_reason_R	50,2%	46,4%	50,0%
		% of Total	48,2%	1,8%	50,0%
	1,00	Count	2036	89	2125
		% within KWIN2	95,8%	4,2%	100,0%
		% within UMN_reason_R	49,8%	53,6%	50,0%
		% of Total	47,9%	2,1%	50,0%
Total		Count	4085	166	4251
		% within KWIN2	96,1%	3,9%	100,0%
		% within UMN_reason_R	100,0%	100,0%	100,0%
		% of Total	96,1%	3,9%	100,0%

a. PRE08 POST14 = 2014

Chi-Square Tests[a]

	Value	df	Asymptotic Significance (2-sided)	Exact Sig. (2-sided)	Exact Sig. (1-sided)
Pearson Chi-Square	,909[b]	1	,340		
Continuity Correction[c]	,764	1	,382		
Likelihood Ratio	,909	1	,340		
Fisher's Exact Test				,343	,191
Linear-by-Linear Association	,908	1	,341		
N of Valid Cases	4251				

a. PRE08 POST14 = 2014

b. 0 cells (,0%) have expected count less than 5. The minimum expected count is 82,98.

c. Computed only for a 2x2 table

```
CROSSTABS
 /TABLES=KWIN3 BY UMN_reason_R
 /FORMAT=AVALUE TABLES
 /STATISTICS=CHISQ
 /CELLS=COUNT ROW COLUMN TOTAL
 /COUNT ROUND CELL.
```

Crosstabs

PRE08 POST14 = 2008

Case Processing Summary[a]

	Cases					
	Valid		Missing		Total	
	N	Percent	N	Percent	N	Percent
KWIN3 * UMN_reason_R	4039	39,9%	6077	60,1%	10116	100,0%

a. PRE08 POST14 = 2008

KWIN3 * UMN_reason_R Crosstabulation[a]

			UMN_reason_R 0	UMN_reason_R 1	Total
KWIN3	,00	Count	1983	35	2018
		% within KWIN3	98,3%	1,7%	100,0%
		% within UMN_reason_R	50,1%	43,8%	50,0%
		% of Total	49,1%	0,9%	50,0%
	1,00	Count	1976	45	2021
		% within KWIN3	97,8%	2,2%	100,0%
		% within UMN_reason_R	49,9%	56,3%	50,0%
		% of Total	48,9%	1,1%	50,0%
Total		Count	3959	80	4039
		% within KWIN3	98,0%	2,0%	100,0%
		% within UMN_reason_R	100,0%	100,0%	100,0%
		% of Total	98,0%	2,0%	100,0%

a. PRE08 POST14 = 2008

Chi-Square Tests[a]

	Value	df	Asymptotic Significance (2-sided)	Exact Sig. (2-sided)	Exact Sig. (1-sided)
Pearson Chi-Square	1,260[b]	1	,262		
Continuity Correction[c]	1,019	1	,313		
Likelihood Ratio	1,263	1	,261		
Fisher's Exact Test				,309	,156
Linear-by-Linear Association	1,260	1	,262		
N of Valid Cases	4039				

a. PRE08 POST14 = 2008

b. 0 cells (,0%) have expected count less than 5. The minimum expected count is 39,97.

c. Computed only for a 2x2 table

PRE08 POST14 = 2014

Case Processing Summary[a]

	Cases Valid N	Valid Percent	Missing N	Missing Percent	Total N	Total Percent
KWIN3 * UMN_reason_R	4252	40,0%	6377	60,0%	10629	100,0%

a. PRE08 POST14 = 2014

KWIN3 * UMN_reason_R Crosstabulation[a]

			UMN_reason_R		
			0	1	Total
KWIN3	,00	Count	2049	77	2126
		% within KWIN3	96,4%	3,6%	100,0%
		% within UMN_reason_R	50,5%	40,3%	50,0%
		% of Total	48,2%	1,8%	50,0%
	1,00	Count	2012	114	2126
		% within KWIN3	94,6%	5,4%	100,0%
		% within UMN_reason_R	49,5%	59,7%	50,0%
		% of Total	47,3%	2,7%	50,0%
Total		Count	4061	191	4252
		% within KWIN3	95,5%	4,5%	100,0%
		% within UMN_reason_R	100,0%	100,0%	100,0%
		% of Total	95,5%	4,5%	100,0%

a. PRE08 POST14 = 2014

Chi-Square Tests[a]

	Value	df	Asymptotic Significance (2-sided)	Exact Sig. (2-sided)	Exact Sig. (1-sided)
Pearson Chi-Square	7,505[b]	1	,006		
Continuity Correction[c]	7,104	1	,008		
Likelihood Ratio	7,550	1	,006		
Fisher's Exact Test				,008	,004
Linear-by-Linear Association	7,503	1	,006		
N of Valid Cases	4252				

a. PRE08 POST14 = 2014

b. 0 cells (,0%) have expected count less than 5. The minimum expected count is 95,50.

c. Computed only for a 2x2 table

```
CROSSTABS
  /TABLES=KWIN4 BY UMN_reason_R
  /FORMAT=AVALUE TABLES
  /STATISTICS=CHISQ
```

```
/CELLS=COUNT ROW COLUMN TOTAL
/COUNT ROUND CELL.
```

Crosstabs

PRE08 POST14 = 2008

KWIN4 * UMN_reason_R Crosstabulation[a]

			UMN_reason_R 0	UMN_reason_R 1	Total
KWIN4	,00	Count	1983	35	2018
		% within KWIN4	98,3%	1,7%	100,0%
		% within UMN_reason_R	49,9%	49,3%	49,9%
		% of Total	49,1%	0,9%	49,9%
	1,00	Count	1988	36	2024
		% within KWIN4	98,2%	1,8%	100,0%
		% within UMN_reason_R	50,1%	50,7%	50,1%
		% of Total	49,2%	0,9%	50,1%
Total		Count	3971	71	4042
		% within KWIN4	98,2%	1,8%	100,0%
		% within UMN_reason_R	100,0%	100,0%	100,0%
		% of Total	98,2%	1,8%	100,0%

a. PRE08 POST14 = 2008

Chi-Square Tests[a]

	Value	df	Asymptotic Significance (2-sided)	Exact Sig. (2-sided)	Exact Sig. (1-sided)
Pearson Chi-Square	,011[b]	1	,915		
Continuity Correction[c]	,000	1	1,000		
Likelihood Ratio	,011	1	,915		
Fisher's Exact Test				1,000	,505
Linear-by-Linear Association	,011	1	,915		
N of Valid Cases	4042				

a. PRE08 POST14 = 2008

b. 0 cells (,0%) have expected count less than 5. The minimum expected count is 35,45.

c. Computed only for a 2x2 table

PRE08 POST14 = 2014

Case Processing Summary[a]

	Cases					
	Valid		Missing		Total	
	N	Percent	N	Percent	N	Percent
KWIN4 * UMN_reason_R	4253	40,0%	6376	60,0%	10629	100,0%

a. PRE08 POST14 = 2014

KWIN4 * UMN_reason_R Crosstabulation[a]

			UMN_reason_R		Total
			0	1	
KWIN4	,00	Count	2049	77	2126
		% within KWIN4	96,4%	3,6%	100,0%
		% within UMN_reason_R	50,1%	47,5%	50,0%
		% of Total	48,2%	1,8%	50,0%
	1,00	Count	2042	85	2127
		% within KWIN4	96,0%	4,0%	100,0%
		% within UMN_reason_R	49,9%	52,5%	50,0%
		% of Total	48,0%	2,0%	50,0%
Total		Count	4091	162	4253
		% within KWIN4	96,2%	3,8%	100,0%
		% within UMN_reason_R	100,0%	100,0%	100,0%
		% of Total	96,2%	3,8%	100,0%

a. PRE08 POST14 = 2014

Chi-Square Tests[a]

	Value	df	Asymptotic Significance (2-sided)	Exact Sig. (2-sided)	Exact Sig. (1-sided)
Pearson Chi-Square	,407[b]	1	,524		
Continuity Correction[c]	,311	1	,577		
Likelihood Ratio	,407	1	,524		
Fisher's Exact Test				,575	,289
Linear-by-Linear Association	,407	1	,524		
N of Valid Cases	4253				

a. PRE08 POST14 = 2014

b. 0 cells (,0%) have expected count less than 5. The minimum expected count is 80,98.

c. Computed only for a 2x2 table

```
CROSSTABS
 /TABLES=KWIN5 BY UMN_reason_R
 /FORMAT=AVALUE TABLES
 /STATISTICS=CHISQ
 /CELLS=COUNT ROW COLUMN TOTAL
 /COUNT ROUND CELL.
```

Crosstabs

PRE08 POST14 = 2008

Case Processing Summary[a]

	Cases					
	Valid		Missing		Total	
	N	Percent	N	Percent	N	Percent
KWIN5 * UMN_reason_R	4040	39,9%	6076	60,1%	10116	100,0%

a. PRE08 POST14 = 2008

KWIN5 * UMN_reason_R Crosstabulation[a]

			UMN_reason_R		Total
			0	1	
KWIN5	,00	Count	1983	35	2018
		% within KWIN5	98,3%	1,7%	100,0%
		% within UMN_reason_R	49,6%	77,8%	50,0%
		% of Total	49,1%	0,9%	50,0%
	1,00	Count	2012	10	2022
		% within KWIN5	99,5%	0,5%	100,0%
		% within UMN_reason_R	50,4%	22,2%	50,0%
		% of Total	49,8%	0,2%	50,0%
Total		Count	3995	45	4040
		% within KWIN5	98,9%	1,1%	100,0%
		% within UMN_reason_R	100,0%	100,0%	100,0%
		% of Total	98,9%	1,1%	100,0%

a. PRE08 POST14 = 2008

Chi-Square Tests[a]

	Value	df	Asymptotic Significance (2-sided)	Exact Sig. (2-sided)	Exact Sig. (1-sided)
Pearson Chi-Square	14,095[b]	1	,000		
Continuity Correction[c]	12,992	1	,000		
Likelihood Ratio	14,916	1	,000		
Fisher's Exact Test				,000	,000
Linear-by-Linear Association	14,092	1	,000		
N of Valid Cases	4040				

a. PRE08 POST14 = 2008

b. 0 cells (,0%) have expected count less than 5. The minimum expected count is 22,48.

c. Computed only for a 2x2 table

PRE08 POST14 = 2014

KWIN5 * UMN_reason_R Crosstabulation[a]

			UMN_reason_R		
			0	1	Total
KWIN5	,00	Count	2049	77	2126
		% within KWIN5	96,4%	3,6%	100,0%
		% within UMN_reason_R	49,6%	63,1%	50,0%
		% of Total	48,2%	1,8%	50,0%
	1,00	Count	2080	45	2125
		% within KWIN5	97,9%	2,1%	100,0%
		% within UMN_reason_R	50,4%	36,9%	50,0%
		% of Total	48,9%	1,1%	50,0%
Total		Count	4129	122	4251
		% within KWIN5	97,1%	2,9%	100,0%
		% within UMN_reason_R	100,0%	100,0%	100,0%
		% of Total	97,1%	2,9%	100,0%

a. PRE08 POST14 = 2014

Chi-Square Tests[a]

	Value	df	Asymptotic Significance (2-sided)	Exact Sig. (2-sided)	Exact Sig. (1-sided)
Pearson Chi-Square	8,626[b]	1	,003		
Continuity Correction[c]	8,095	1	,004		
Likelihood Ratio	8,725	1	,003		
Fisher's Exact Test				,004	,002
Linear-by-Linear Association	8,624	1	,003		
N of Valid Cases	4251				

a. PRE08 POST14 = 2014

b. 0 cells (,0%) have expected count less than 5. The minimum expected count is 60,99.

c. Computed only for a 2x2 table
